# Supplementary material for: Endogenous Glycoprotein GPM6a Is Involved in Neurite Outgrowth in Rat Dorsal Root Ganglion Neurons
Source: Biomolecules. 2023 Mar 25;13(4):594. doi: 10.3390/biom13040594 (PMC10136334; doi:10.3390/biom13040594)
Supplement: Supplementary file 1 [file biomolecules-13-00594-s001.zip › biomolecules-2238135-supplementary/biomolecules-2238135-supplementary-to final.pdf]

Supplementary figures and legends:

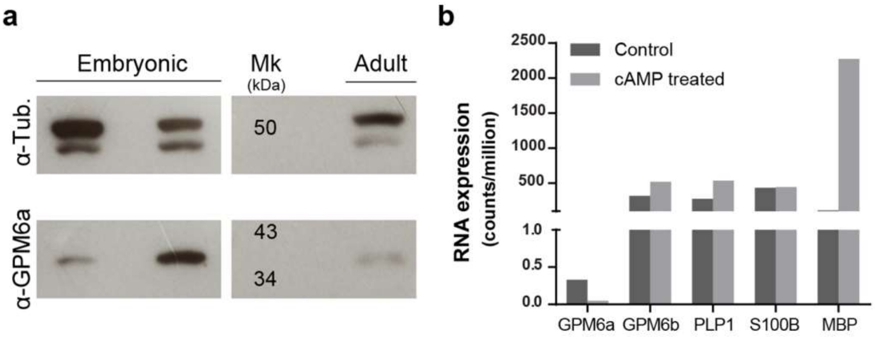

Figure S1: GPM6a is detected in DRGs homogenates but not in SCs. **(a)** Representative Western blot of rat embryonic and adult DRGs. Primary antibodies against GPM6a and Tubulin (loading control) were used to assess the expression of GPM6a in undissociated, uncultured rat DRG tissue. Mk: molecular weight marker. **(b)** RNA-seq analysis of pure rat SCs in culture, treated or not with cAMP, as described in our prior work ([38]). The graph shows the expression of GPM6a transcripts as compared to other members of the PLP family (GPM6b and PLP1) and the SC markers, S100β, and MBP (a myelin protein).

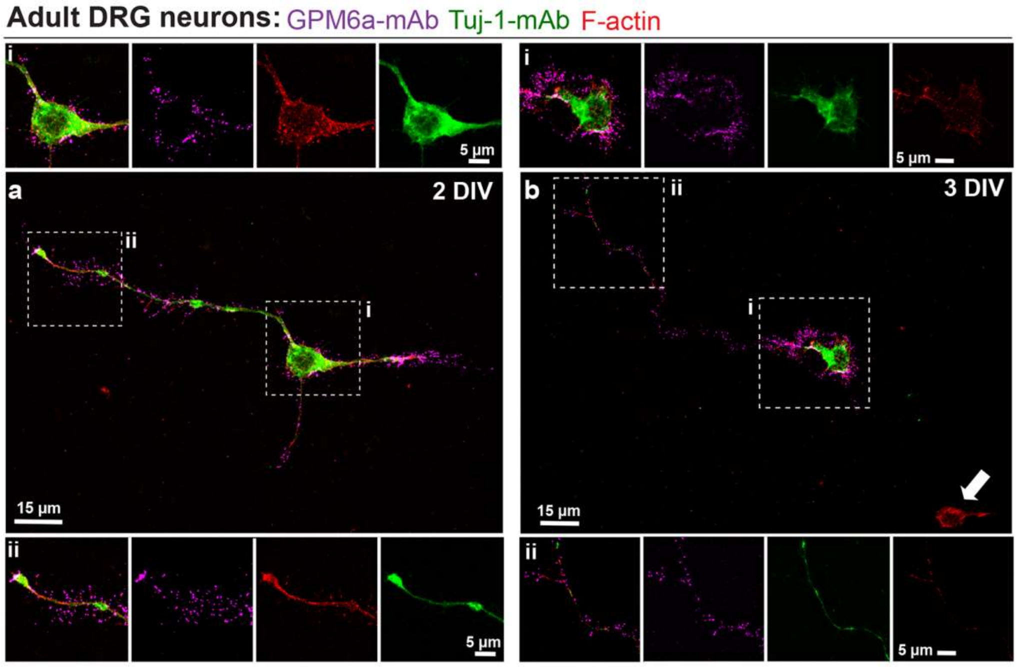

Figure S2: GPM6a is detected in a punctate pattern in the soma and processes of adult DRG neurons in culture**.** Representative images of adult dissociated cultured DRG neurons at 2 DIV (a) and 3 DIV (b) are shown. N=2-3 independent experiments and n=10-20 cells per condition (days *in vitro*) were analyzed. Each experiment had at least three wells per condition (n=3 technical replicates). Images show positive immunostaining for GPM6a in magenta (GPM6a-mAb), β-Tubulin III in green (Tuj-1-mAb), and actin in red (F-actin). GPM6a is detected within the soma (insets a-i and b-i) and the neuronal processes (insets a-ii and b-ii). White arrow in (b) indicates a representative non-neuronal cell (negative for β-Tubulin III) where no staining for GPM6a was detected. Confocal images were acquired with an Olympus FV1000 confocal microscope, using a 60X objective with no digital zoom. Scale bars are indicated in the figure.

Supplementary videos legends:

Video S1: GPM6a´s detection in embryonic rat DRGs**.** Representative video of an embryonic DRG where positive immunostaining for GPM6a is shown in magenta. β-III tubulin is shown in green, and the cell nuclei are shown in cyan. Intact DRGs were fixed, prepared for sectioning on a cryostat (at 20 μm), and analyzed immunohistochemically. Confocal images were acquired with an Olympus FV1000 confocal microscope using a 60X objective and a 2X digital zoom. The video is a reconstruction of 31 z-stacks using 0.25 μm step size for each z-stack (total depth = 7.75 μm). The scale bar is indicated in the video.

Video S2: GPM6a´s detection in adult rat DRGs**.** Representative video of an adult DRG where positive immunostaining for GPM6a is shown in magenta. β-Tubulin III is shown in green, and the cell nuclei are shown in cyan. Intact DRGs were fixed, prepared for sectioning on a cryostat (at 20 μm), and analyzed immunohistochemically. Confocal images were acquired with an Olympus FV1000 confocal microscope using a 60X objective and a 1X digital zoom was used. The video is a reconstruction of 29 z-stacks using 0.5 μm step size for each z-stack (total depth = 14.5 μm). The scale bar is indicated in the video.
