# Supplementary figures and images for: Endogenous Glycoprotein GPM6a Is Involved in Neurite Outgrowth in Rat Dorsal Root Ganglion Neurons
Source: Biomolecules. 2023 Mar 25;13(4):594. doi: 10.3390/biom13040594 (PMC10136334; doi:10.3390/biom13040594)

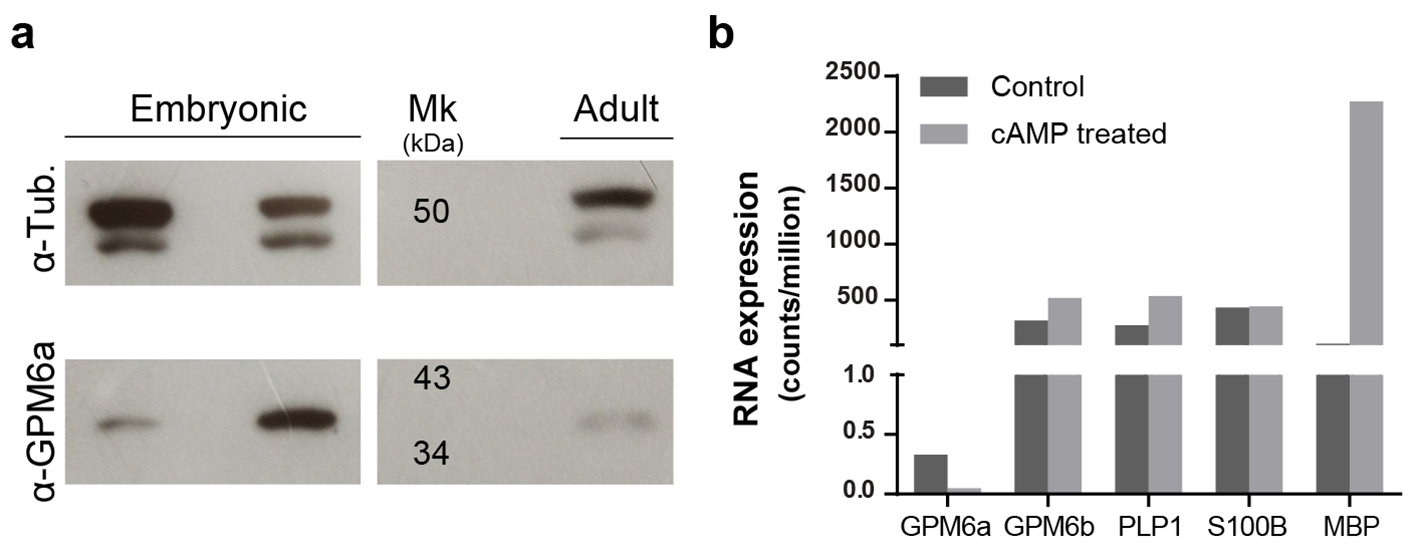

Supplement: Supplementary file 1 [file biomolecules-13-00594-s001.zip › biomolecules-2238135-supplementary/Suplementary figures/Figure S1.tif]

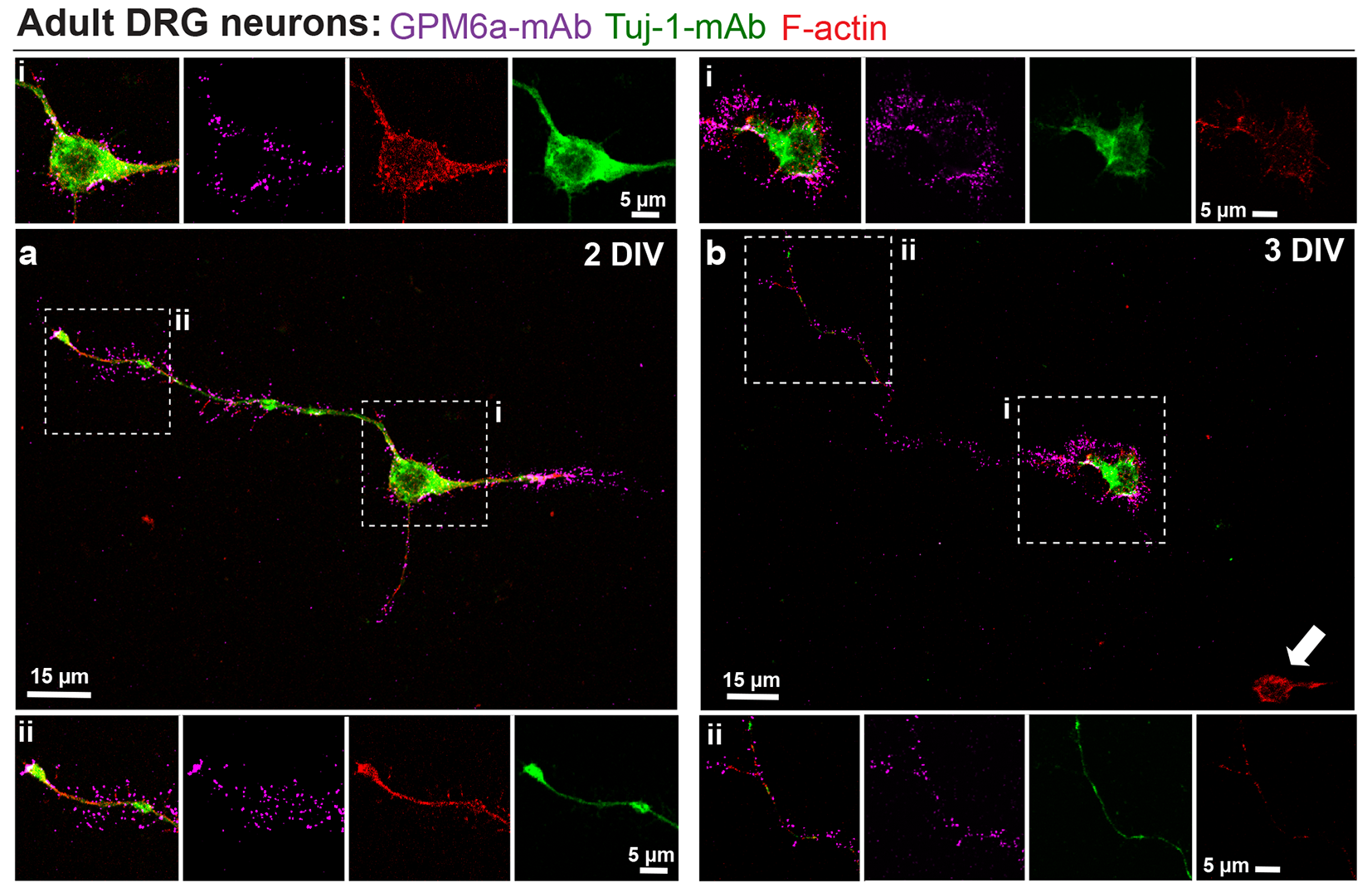

Supplement: Supplementary file 1 [file biomolecules-13-00594-s001.zip › biomolecules-2238135-supplementary/Suplementary figures/Figure S2.tif]
